# Medical Ethics

**Published:** 1856-12

**Authors:** 


*Medical Ethics.*

In a former number of the *Journal* we made some comments on a hand-bill, purporting to come from Drs. Kreider & Kimber, of Prairie City. We have since been satisfactorily assured, that though the hand-bill was printed, it was never actually circulated or distributed among the people, simply because its authors, on reading it over, were satisfied that it was not in accordance with the Code of Ethics, or with that sense of propriety which should govern every intelligent physician.

We are glad to make this correction, because our previous acquaintance with Dr. Kreider had led us to regard him as a high-minded and honorable man in all the relations of life, and we would not, knowingly, do him or any other the slightest injustice.

